# Longitudinal serum S100β and brain aging in the Lothian Birth Cohort 1936

**DOI:** 10.1016/j.neurobiolaging.2018.05.029

**Published:** 2018-09

**Authors:** Simon R. Cox, Mike Allerhand, Stuart J. Ritchie, Susana Muñoz Maniega, Maria Valdés Hernández, Sarah E. Harris, David Alexander Dickie, Devasuda Anblagan, Benjamin S. Aribisala, Zoe Morris, Roy Sherwood, N. Joan Abbott, John M. Starr, Mark E. Bastin, Joanna M. Wardlaw, Ian J. Deary

**Affiliations:** a Centre for Cognitive Ageing and Cognitive Epidemiology, University of Edinburgh, Edinburgh, Scotland, UK; b Department of Psychology, University of Edinburgh, Edinburgh, Scotland, UK; c Department of Neuroimaging Sciences, Centre for Clinical Brain Sciences, University of Edinburgh, Edinburgh, Scotland, UK; d UK Dementia Research Institute at The University of Edinburgh, Edinburgh, UK; e Medical Genetics Section, University of Edinburgh Centre for Genomic and Experimental Medicine and MRC Institute of Genetics and Molecular Medicine, Western General Hospital, Edinburgh, UK; f Institute of Cardiovascular and Medical Sciences College of Medical, Veterinary & Life Sciences University of Glasgow, UK; g Department of Computer Science, Lagos State University, Lagos, Nigeria; h Department of Clinical Biochemistry, King's College Hospital NHS Foundation Trust, London, UK; i Institute of Pharmaceutical Science, King's College London, London, UK; j Alzheimer Scotland Dementia Research Centre, University of Edinburgh, Edinburgh, Scotland, UK

**Keywords:** S100β, White matter, Small vessel disease, Aging, Longitudinal

## Abstract

Elevated serum and cerebrospinal fluid concentrations of S100β, a protein predominantly found in glia, are associated with intracranial injury and neurodegeneration, although concentrations are also influenced by several other factors. The longitudinal association between serum S100β concentrations and brain health in nonpathological aging is unknown. In a large group (baseline N = 593; longitudinal N = 414) of community-dwelling older adults at ages 73 and 76 years, we examined cross-sectional and parallel longitudinal changes between serum S100β and brain MRI parameters: white matter hyperintensities, perivascular space visibility, white matter fractional anisotropy and mean diffusivity (MD), global atrophy, and gray matter volume. Using bivariate change score structural equation models, correcting for age, sex, diabetes, and hypertension, higher S100β was cross-sectionally associated with poorer general fractional anisotropy (*r* = −0.150, *p* = 0.001), which was strongest in the anterior thalamic (*r* = −0.155, *p* < 0.001) and cingulum bundles (*r* = −0.111, *p* = 0.005), and survived false discovery rate correction. Longitudinally, there were no significant associations between changes in brain imaging parameters and S100β after false discovery rate correction. These data provide some weak evidence that S100β may be an informative biomarker of brain white matter aging.

## 1 Introduction

The calcium-binding protein S100β has clinical value as a proteomic biomarker of central nervous system damage. It is primarily found in glial cells, but also in some neuronal populations and in melanocytes, among other cell types (Donato, 2006, Donato et al., 2013). At nanomolar concentrations, S100β exerts neuroprotective and neurotrophic influences, but elevated S100β may contribute to further negative effects, as its presence at micromolar concentrations increases expression of proinflammatory cytokines, leading to apoptosis (Kleindienst et al., 2010, Rothermundt et al., 2003, Steiner et al., 1999). S100β is elevated after traumatic brain injury in both cerebrospinal fluid (CSF) and serum (Ingebrigtsen and Romner, 2002, Petzold et al., 2003, Vos et al., 2010), with greater S100β concentrations prognostic of poorer outcomes and recovery (Goyal et al., 2013, Thelin et al., 2017). Serum S100β levels are also influenced by blood-brain barrier (BBB) leakage (Kapural et al., 2002, Kleindienst et al., 2010, Ucar et al., 2004), as well as from other sources such as bone fractures, exercise, muscle injury, burns and melanoma (Anderson et al., 2001, Harpio and Einarsson, 2004, Koh and Lee, 2014, Mocellin et al., 2008, Mohammed et al., 2001, Pelinka et al., 2003).

Although S100β has been investigated as a biomarker (in serum and CSF) in studies of head injury, depression, and neurodegenerative diseases such as Alzheimer's disease (Chaves et al., 2010, Peskind et al., 2001, Polyakova et al., 2015), the neurostructural correlates of S100β and its longitudinal trajectories in nonpathological aging are underinvestigated. S100β concentrations are positively associated with age (Nygaard et al., 1997, Schroeter et al., 2011, van Engelen et al., 1992), although some (Portela et al., 2002, Wiesmann et al., 1998) found no age effect in adulthood. Identifying possible biomarkers of brain aging is a key challenge (Academy of Medical Sciences, 2016, Jylhävä et al., 2017), and serum S100β is one of the logical candidates, yet data on S100β and multimodal brain analyses in older participants are lacking. Two prior cross-sectional studies indicate that serum S100β is specifically associated with poorer white matter microstructure (assessed with diffusion tensor MRI) in a small sample of healthy participants (N = 41, effect found in females only; Streitbürger et al., 2012), and in a small study of schizophrenia patients versus controls (total N = 39; Milleit et al., 2016). Neither study found a significant association between S100β and gray matter (GM)—however, it should be noted that both adopted a voxel-based–morphometry approach which results in reduced power in the large areas of the cortex that show highly individualized patterns of gyrification, and insensitivity to discrete lesions; Tisserand et al., 2004). Another study (N = 102; van der Leeuw et al., 2017) found no association between S100β and either white matter fractional anisotropy (FA) or cortical thickness in a mixed sample of patients with psychosis, relatives, and controls. Thus, well-powered, longitudinal, multimodal imaging studies—in participants at an age that confers relatively high risk of brain structural decline—are required to examine the possible differential sensitivity of S100β to cross-sectional levels of, and longitudinal declines in, various imaging parameters and brain tissues.

Other candidate MRI parameters that may relate to S100β are markers of cerebral small vessel disease (SVD) burden. There is increasing evidence that BBB leakage occurs as an underlying pathology in SVD (Wardlaw et al., 2017, Zhang et al., 2017). The presence of white matter hyperintensities (WMHs) and perivascular spaces (PVS) are important markers of SVD pathophysiology that increase with age, in cerebrovascular disease, are linked to increased risk of stroke, and are associated with both cognitive impairment and dementia (Doubal et al., 2010, Wardlaw et al., 2015). PVS are also associated with elevated plasma markers of inflammation in older participants (Aribisala et al., 2014). They are also more frequent in patients with lacunar stroke and WMH, and are more visible with increasing evidence of BBB leakage in patients with SVD-related stroke (Wardlaw et al., 2009). PVS are also more visible with inflammation and BBB leakage in active multiple sclerosis plaques (Wuerfel et al., 2008). Although plasma S100β is influenced by BBB leakage in head injury as well as general cerebral pathology, it remains unknown whether S100β is associated with these important SVD markers on brain MRI in healthy community-dwelling older adults.

In the present study, we investigated the level and change in S100β and indices of structural and diffusion MRI, in a large cohort of older individuals measured at ages 73 and 76 years. Given that S100β concentration in blood may rise due to age, central nervous system (CNS) damage, and BBB disruption, we hypothesized that relatively higher and increasing concentrations of plasma S100β would be coupled with lower and decreasing measures of brain structural and microstructural health. Prior evidence indicates that S100β is particularly strongly expressed in the human brain's white matter tracts (histological data showed co-localization of S100β with oligodendrocyte markers in the human brain; Streitbürger et al., 2012), and that serum S100β is cross-sectionally associated with poorer white matter microstructure (assessed with diffusion tensor MRI) in small mixed samples (N ≤ 102) with wide age ranges (Milleit et al., 2016, Streitbürger et al., 2012). Thus, we hypothesize that elevated and increasing serum S100β would be particularly pertinent to poorer and decreasing white matter structure, beyond measures of global atrophy and GM volume.

## 2 Materials and methods

### 2.1 Participants

Data are drawn from waves 2 and 3 of a longitudinal study of aging: the Lothian Birth Cohort 1936 study (LBC1936; Deary et al., 2007, Deary et al., 2012), when participants were a mean age of about 73 and 76 years, respectively. In 1947, Scotland tested the intelligence of almost all schoolchildren born in 1936, and the LBC1936 follows up some of those individuals—now in older age—who mostly live in the Edinburgh and Lothians area. The initial wave of LBC1936 (wave 1) took place between 2004 and 2007. It assessed 1091 individuals on aspects of their health, and physical and cognitive function, at around 70 years old (M = 69.53, SD = 0.832). At wave 2 (2007–2011) and wave 3 (2011–2013), 866 and 697, respectively, returned at mean ages of about 73 and 76 years, a detailed MRI brain scan was added to the protocol at both waves (Wardlaw et al., 2011). During a medical interview at each wave, participants reported their medical history (including a self-reported diagnosis of hypertension, diabetes, melanoma, and dementia). The Multi-Centre Research Ethics Committee for Scotland (MREC/01/0/56), the Scotland A Research Ethics Committee (07/MRE00/58), and the Lothian Research Ethics Committee (LREC/2003/2/29) approved the use of the human participants in this study; all participants provided written informed consent and these have been kept on file.

### 2.2 S100β

Serum samples were obtained from participants during the main physical and cognitive testing appointment at waves 2 and 3. The mean lag between waves was 3.77 years (SD = 0.28). After collection, samples were stored at −80 °C at the Wellcome Trust Clinical Research Facility, Western General Hospital, Edinburgh, until the conclusion of the wave. They were then transferred to the Department of Clinical Biochemistry, King's College London using cold-chain logistics, where they were stored at −20 °C until assays were conducted using a chemiluminescence immunoassay S100β kit (distributed by DiaSorin, Berks, UK) on a LIAISON chemiluminescence analyzer. The lag between sample dispatch at the end of sampling and assay completion (i.e., time stored at −20 °C rather than −80 °C) was an average of 44 days (SD = 26) for 4 batches at wave 2, and 8 days (single batch) at wave 3, respectively. The minimal detectable concentration of the assay was 0.02 μg/L. Intra- and inter-assay precision for both waves is reported in Supplementary Table A.1.

### 2.3 MRI acquisition and processing

Participants underwent whole-brain structural and diffusion MRI using the same 1.5 T GE Signa Horizon scanner (General Electric, Milwaukee, WI, USA) at wave 2 and 3. The scanner is maintained with a careful quality control programme. Scans took place at the Brain Research Imaging Centre, Edinburgh, shortly after serum collection (mean lag for the present study sample: wave 2 M = 65.39 days, SD = 34.69; wave 3 M = 38.69 days, SD = 28.37). Full details of acquisition and processing are available in an open access protocol article (Wardlaw et al., 2011). Briefly, T_1_-, T_2_-, T_2_*-, and FLAIR-weighted sequences were co-registered (voxel size = 1 × 1 × 2 mm). Total brain (TB), GM, and white matter hyperintensity volumes were quantified using a semiautomated multispectral fusion method (Valdés Hernández et al., 2010). WMHs were explicitly defined as punctate, focal, or diffuse lesions in subcortical regions and distinguished from lacunes and PVS by signal characteristics (Wardlaw et al., 2013). Cortical or discrete subcortical infarcts were excluded by careful manual editing blind to other features. PVS were defined as fluid-containing small spaces running parallel with the expected direction of perforating vessels, appearing punctate in cross section and linear in longitudinal section, with <3 mm diameter. PVS were differentiated from lacunes or WMH on morphology, signal, and size criteria as previously defined (Wardlaw et al., 2013, Potter et al., 2015a). From the T2-weighted volumes, PVS ratings were performed by a trained neuroradiologist (JMW, ZM; Potter et al., 2015a, Potter et al., 2015b). Change in PVS between waves was scored by comparing scans at wave 2 and wave 3 side by side, blind to any other participant characteristics, and scored on a 5-point scale from −2 (reduction, i.e., improvement) to +2 (increase, i.e., worsening), where 0 denotes no visible change.

The diffusion tensor MRI (DT-MRI) acquisition comprised a single-shot spin-echo echo-planar diffusion weighted volumes (*b* = 1000 s mm^−2^) acquired in 64 noncollinear directions, alongside 7 T_2_-weighted images (*b* = 0 s mm^−2^). This yielded 72 contiguous axial slices (FoV = 256 × 256 mm, matrix 128 × 128, 2 mm isotropic voxels). Repetition and echo times were 16.5 s and 95.5 ms, respectively. After preprocessing (brain extraction, removal of bulk participant motion and eddy current–induced distortions), water diffusion tensor parameters were estimated using FSL tools (FMRIB; Oxford, UK; http://www.fmrib.ox.ac.uk). A 2-fiber model with 5000 streamlines was then used to create brain connectivity data using the BEDPOSTX/ProbTrackX algorithm in 12 tracts of interest: the genu and splenium of the corpus callosum, bilateral anterior thalamic radiation, cingulum, uncinate, arcuate, and inferior longitudinal fasciculi. Probabilistic neighborhood tractography as implemented in the TractoR package (http://www.tractor-mri.org.uk; Clayden et al., 2011) identified the tracts of interest from the connectivity data (Bastin et al., 2010, Muñoz Maniega et al., 2017). White matter tract-averaged FA and mean diffusivity (MD) were then derived as the average of all voxels contained within the resultant tract maps. All segmented images were visually inspected for accuracy, blind to participant characteristics, to identify and correct errors.

### 2.4 Statistical analysis

We excluded from the analyses those with self-reported history of dementia, or Mini-Mental State Examination score of <24 (n = 35). This was based on prior reports of elevated S100β in dementia (Chaves et al., 2010), the likelihood that these individuals were undergoing pathological CNS degeneration, and their low numbers in the current cohort (yielding low statistical power with which to reliably detect associations). Given that elevated serum S100β is associated with melanoma (Harpio and Einarsson, 2004, Mocellin et al., 2008, Mohammed et al., 2001), those who reported melanoma at either wave (n = 26) were also excluded. S100β concentrations for excluded participants are shown in Supplementary Table A.2. Both WMH measures were log transformed to correct skewness. Extreme outlying points (>4 SDs above the mean) for S100β at wave 2 (n = 6) and wave 3 (n = 4) were removed, along with 7 points at wave 3 that were below the sensitivity threshold of the assay (<0.02 μg/L). After exclusions, a total of 776 and 619 participants provided S100β data at ages 73 and 76 years, respectively, 593 and 414 of whom also provided brain MRI data. We used the maximum available sample size in all analyses.

**Fig. 1:**
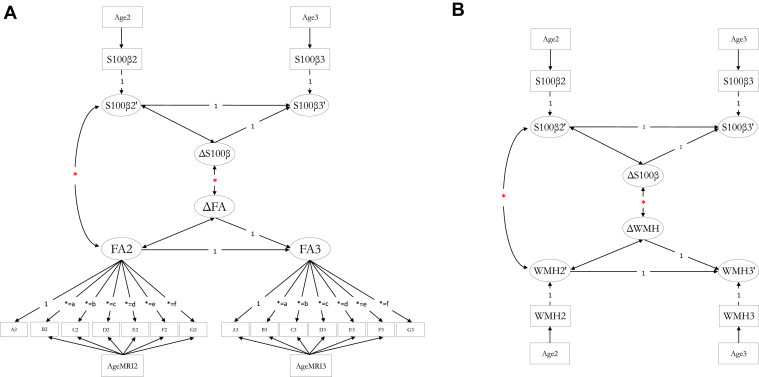
Example of bivariate change score models. Baseline level of, and 3-year change in, S100β is associated with the wave 2 level and wave 2–wave 3 (age 73–76 years) change in (A) a latent factorially invariant measure of FA, and (B) white matter hyperintensity volume. Individual tract-averaged values in (A) are A:G (correlated residuals not shown). For PVS analysis, the visual rating of change replaces the MRI-based delta (ΔWMH, in this example). Manifest (observed) variables are corrected for age in days at serum collection at both waves (Age2, Age3), and at the 2 MRI scans (AgeMRI2, AgeMRI3), which corrects for within-wave lag between serum and MRI collection. All observed variables are also corrected for sex (not shown), with MRI data also corrected for diabetes and hypertension diagnoses (not shown). * denotes cross-sectional and longitudinal relations of interest. Abbreviations: FA, fractional anisotropy; PVS, perivascular space rating; WMH, white matter hyperintensity. (For interpretation of the references to color in this figure legend, the reader is referred to the Web version of this article.)

The main questions we addressed were (1) are there associations between serum S100β and brain imaging variables cross-sectionally at age 73 years? and (2) are the changes in S100β from age 73 years to age 76 years correlated with changes in brain imaging variables across the same ages? We used bivariate change score models (McArdle, 2009) in a structural equation modeling (SEM) framework to test these cross-sectional and longitudinal associations between S100β and brain MRI variables (Fig. 1), specifying a separate model for each brain MRI outcome. In the case of PVS analysis, the visual rating of PVS change was used in place of a latent change score, and correlated with the latent S100β change score. The volumetric brain indices were expressed as a proportion of intracranial volume in our main SEMs, and we also provided a supplementary analysis for uncorrected measures. Using the FA and MD measures across multiple white matter tracts, we derived a latent variable (hereafter referred to as *g*FA and *g*MD) for waves 2 and 3, respectively, using the following: genu and splenium of the corpus callosum, and left-right averages of the anterior thalamic radiation, inferior longitudinal fasciculus, uncinate, arcuate, and cingulum. We imposed strong factorial invariance (as was previously shown to be possible for these data; Ritchie et al., 2015), constraining the intercepts of each tract measure and their loadings on the latent variable to equality across waves. We also included correlated residuals between corresponding tracts across waves, alongside 5 other significant tract-tract residual paths for *g*FA and 6 for *g*MD. This builds on our and others' prior work, which found that there is substantial shared variance in white matter microstructural properties across tracts of the brain in early life, middle, and older age (Cox et al., 2016, Penke et al., 2010, Ritchie et al., 2015, Telford et al., 2017). Thus, these general, latent, factors reflect common microstructural properties (FA and MD) across white matter pathways. Finally, based on evidence of local white matter variation in S100β expression (most strongly expressed in the corpus callosum and cingulum bundle) and cross-sectional associations between FA and S100β (Streitbürger et al., 2012, Milleit et al., 2016, van der Leeuw et al., 2017, Allen Institute for Brain Science, 2010), we used the same framework as above to examine associations between S100β and tract-specific microstructure in each white matter tract of interest for FA and MD.

Given that there was a short delay between serum collection and MRI scanning at both waves, we corrected MRI and S100β for their respective age in days at data collection within each model, along with sex, diabetes, and hypertension. To account for missing data bias due to attrition between waves, we took account of all available data, using full information maximum likelihood estimation. We assessed model fit according to the *χ*^2^ minimum function test statistic, the root mean square error of approximation (RMSEA), comparative fit index (CFI), Tucker-Lewis index (TLI), and the standardized root mean square residual (SRMR). All statistical analyses were conducted in R version 3.2.2 “Fire Safety” (R Core Team, 2015). SEM was conducted with the “lavaan” package (Rosseel, 2012) and the resultant *p*-values for the associations of interest (see asterisks in Fig. 1) were corrected for multiple comparisons with false discovery rate (FDR; Benjamini and Hochberg, 1995) using the “p.adjust” function in R.

## 3 Results

### 3.1 Participant and S100β descriptives, and analysis of losses to follow-up

**Fig. 2:**
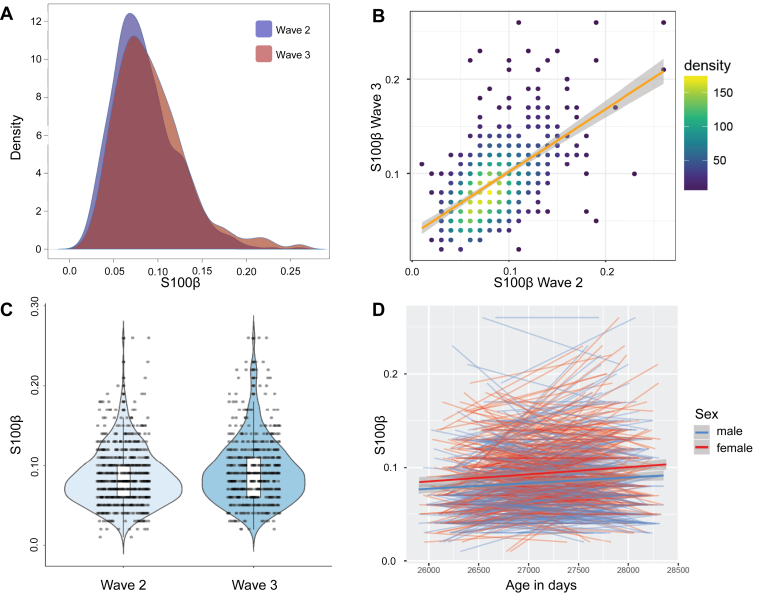
Density plot (A), scatterplot with regression line and 95% CIs (B), beanplot to show mean differences (points jittered for visualization) (C), and plot of individual profiles of returners at Wave 3, colored by sex, to illustrate individual differences in trajectories of S100β (μg/L) change (D). (For interpretation of the references to color in this figure legend, the reader is referred to the Web version of this article.)

Participant characteristics are shown in Table 1, and bivariate associations among study variables are reported in Supplementary Table A.3. Descriptive plots of S100β (density, and age 73–76 years correlation, boxplot, and trajectory) are in Fig. 2. S100β concentrations showed substantial stability of individual differences from age 73 years to age 76 years (Pearson's *r* = 0.585, *p* < 0.001). S100β concentrations were significantly higher at age 76 years than at age 73 years (when considering returners only: wave 2 S100β M = 0.085, SD = 0.035; wave 3 S100β M = 0.092, SD = 0.040; *t* (1190.70) = 3.244, *p* = 0.001, Cohen's *d* = 0.118). Males showed lower S100β than females at both waves (wave 2: *t* (773.28) = 3.655, *p* < 0.001; wave 3: *t* (614.69), *p* = 0.002), but did not exhibit differences in their rate of change with age (*p* = 0.546; Fig. 2).

**Table 1:** Participant characteristics

Variable	Units	Wave 2 (age 73 y)		Wave 3 (age 76 y)	
		Mean (SD)	N	Mean (SD)	N
Sex	M:F	395:381	776	314:305	619
Age S100β	Years	72.493 (0.716)	776	76.243 (0.679)	619
S100β	μg/L	0.085 (0.035)	776	0.092 (0.040)	619
MMSE	/30	28.815 (1.285)	775	28.838 (1.291)	619
WMH	cm^3^	8.060 (11.376)^a^	592	10.998 (15.542)^a^	413
Total Brain	cm^3^	991.176 (90.522)	593	976.179 (91.483)	414
GM	cm^3^	472.862 (44.860)	593	466.321 (43.753)	411

Descriptive MRI data are provided for those that gave S100β at the same wave.

Key: GM, gray matter volume; MMSE, Mini-Mental State Examination; WMH, white matter hyperintensity volume (untransformed).

a Median and IQR provided for raw (untransformed) volumes.

When comparing baseline values of those who returned to provide an S100β sample at age 76 years from those who did not, there were no significant differences for S100β (*t* (270.19) = 0.115, *p* = 0.908), TB volume (*t* (167.74) = 1.577, *p* = 0.117), WMH volume (*t* (152.70) = 1.277, *p* = 0.204). However, individuals who returned at age 76 years had significantly more GM at age 73 years than nonreturners (*t* (174.00) = 2.800, *p* = 0.006). To satisfy the assumption of missing at random (MAR; Rubin, 1976), under which FIML operates, baseline GM volume was included as an auxiliary variable (Schafer and Graham, 2002) when modeling associations between S100β and all other imaging variables. Significant increases in WMH volume and white matter tract MD, and significant decreases in TB volume, GM volume, and white matter FA exhibited by this cohort between wave 2 and 3 have been previously reported elsewhere (Dickie et al., 2016, Ritchie et al., 2015). In the context of the current sample, all brain measures showed statistically significant mean changes over time, considering only those who provided scans at both waves. There were significant reductions in raw TB (*t* (851.73) = 2.815, *p* = 0.005, Cohen's *d* = 0.191) and GM volume (*t* (844.37) = 2.861, *p* = 0.004, Cohen's *d* = 0.197), and increases in WMH volumes (*t* (809.50) = −4.267, *p* < 0.001, Cohen's *d* = 0.293). The visual ratings of PVS change across waves showed that PVS load either stayed consistent (N = 426 received a score of 0) or became worse over time (N = 42 received a score of +1). Those who provided S100β and did not undergo an MRI scan were not significantly different from those who provided both—at either wave 2 or wave 3— in terms of age, S100β concentrations, and Mini-Mental State Examination score (all *t* values <0.779, *p* > 0.437). However, those who undertook both elements of the study comprised a significantly larger proportion of males at wave 2 (*χ*^2^ = 5.708, *p* = 0.017), although not at wave 3 (*χ*^2^ = 2.303, *p* = 0.129).

### 3.2 Cross-sectional and longitudinal associations between S100β and global MRI

Individuals showed substantial variation in the degree to which S100β and the continuous MRI indices changed over time, as indicated by significant slope variances (all *p* < 0.001); slope means and variances from age- and sex-corrected univariate change score models are reported in Supplementary Table A.4. Results of the SEM analyses are shown in Table 2, with bias-corrected 95% confidence intervals from 1000 bootstraps. Model fit statistics are shown in Supplementary Table A.5. Models examining associations between the level and change of S100β and volumetric MRI indices showed adequate fit to the data (WMH: *χ*^2^(28) = 42.629, RMSEA = 0.023, CFI = 0.993, TLI = 0.988, SRMR = 0.026; GM: *χ*^2^(24) = 42.337, RMSEA = 0.027, CFI = 0.987, TLI = 0.977, SRMR = 0.022; TB volume: *χ*^2^(28) = 131.237, RMSEA = 0.060, CFI = 0.931, TLI = 0.887, SRMR = 0.039). None of these measures showed significant cross-sectional associations with S100β at age 73 years (all absolute *r*-values ≤0.061, all *p*-values ≥0.113) or longitudinally (all absolute *r*-values ≤0.082, all *p*-values ≥0.095). Running these volumetric analyses without correction for intracranial volume did not substantially alter the results (Supplementary Table A.6). The model of visually rated PVS change showed an adequate fit to the data (*χ*^2^(14) = 39.439, RMSEA = 0.042, CFI = 0.963, TLI = 0.936, SRMR = 0.032). There was no association between S100β at age 73 years and visually rated PVS change (*r* = −0.034, *p* = 0.475), and the nominally significant association with longitudinal S100β concentrations (*r* = −0.096, *p* = 0.041) did not survive FDR correction.

**Table 2:** Cross-sectional (age 73 y) and longitudinal (age 73 y to age 76 y) associations between S100β and MRI variables

Variable	Cross-sectional (age 73 y)	95% CI		Longitudinal (age 73–76 y)	95% CI	
		Lower	Upper		Lower	Upper
WMH^a^	−0.019 (0.634)	−0.092	0.074	0.082 (0.095)	0.002	0.174
PVS	−0.034 (0.475)^b^	−0.132	0.050	−0.096 (0.041)^b^	−0.183	−0.007
*g*FA	−**0.150 (0.001)**	−0.233	−0.065	−0.083 (0.154)	−0.199	0.038
*g*MD	0.003 (0.941)	−0.099	0.114	−0.019 (0.717)	−0.135	0.075
GM	0.061 (0.133)	−0.021	0.127	−0.050 (0.309)	−0.138	0.049
TBV	0.045 (0.272)	−0.024	0.125	−0.044 (0.364)	−0.136	0.044

Data are shown as standardized coefficients (*p* values), with bootstrapped 95% confidence intervals (CIs) from 1000 draws.

Bold text indicates FDR *q* < 0.05.

Key: FDR, false discovery rate; *g*FA, general factor of white matter tract fractional anisotropy; *g*MD, general factor of white matter tract mean diffusivity; GM, gray matter volume; PVS, perivascular space rating; TBV, total brain volume; WMH, white matter hyperintensity volume.

a log transformed.

b coefficients are for associations between visually rated PVS change (rather than a latent change score) with S100β level and change.

The models examining associations of S100β with white matter diffusion parameters both fitted the data well (*g*FA: *χ*^2^(205) = 284.004, RMSEA = 0.019, CFI = 0.977, TLI = 0.969, SRMR = 0.035 and *g*MD: *χ*^2^(205) = 322.817, RMSEA = 0.024, CFI = 0.970, TLI = 0.959, SRMR = 0.048); tract loadings are reported in Supplementary Table A.7. At wave 2, higher S100β was significantly associated with “less healthy” white matter *g*FA (i.e., poorer directional coherence of water molecular diffusion; *r* = −0.150, *p* = 0.001), which survived correction for multiple comparisons. The 3-year association between declining *g*FA and increasing S100β was nonsignificant (*r* = −0.083, *p* = 0.154). Associations between *g*MD and S100β were nonsignificant for both level (*r* = 0.003, *p* = 0.941) and change (*r* = 0.019, *p* = 0.717).

### 3.3 Cross-sectional and longitudinal associations between S100β and white matter tract-specific microstructure

**Fig. 3:**
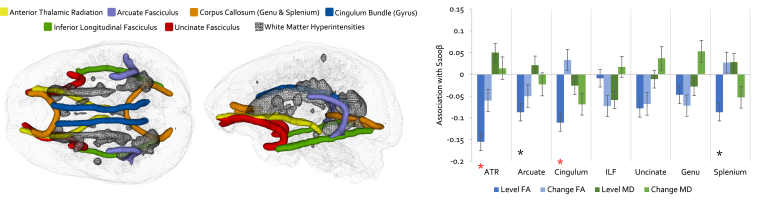
Cross-sectional and longitudinal associations between S100β and white matter tract microstructure. Left panel shows superior and lateral views of tracts of interest (with WMH for illustration) using MRI data from a single LCB1936 participant. Right panel indicates the association magnitude (*r*) for S100β-tract (FA and MD) associations at baseline (cross-sectional at age 73 years; dark blue and dark green) and coupled 3 years change (longitudinal changes in S100β and microstructure from age 73 to 76 years; light blue and light green), with standard error bars. Black and red asterisks denote nominal (*p* < 0.05) and FDR-corrected significance, respectively. Estimates and *p*-values are reported in Supplementary Tables A.9 and A.10. Abbreviations: FA, fractional anisotropy; FDR, false discovery rate; MD, mean diffusivity; WMH, white matter hyperintensity. (For interpretation of the references to color in this figure legend, the reader is referred to the Web version of this article.)

Next, we examined the level and change associations between S100β and average white matter microstructure (FA and MD) within each of the tracts of interest. Fit statistics indicated that all models fitted the data well (Supplementary Tables A.8 and A.9). Results of the models are shown in Fig. 3, and Supplementary Tables A.10 and A.11. A higher concentration of S100β at age 73 years was significantly associated with “poorer” FA at the same age in the anterior thalamic radiation (*r* = −0.155 *p* < 0.001) and cingulum bundle (*r* = −0.111 *p* = 0.005). Both survived FDR correction. There were also nominally significant associations with the level of the splenium (*r* = −0.087 *p* = 0.030) and arcuate (*r* = −0.087 *p* = 0.032) in the same direction, but these did not survive multiple comparison correction. The corresponding associations for tract MD were all nonsignificant cross-sectionally (all absolute *r* values ≤0.059 *p*-values ≥0.138) and longitudinally (all absolute *r* values ≤0.069 *p*-values ≥0.158).

## 4 Discussion

These data represent the first large-scale study of longitudinal S100β concentrations and their association with longitudinal multimodal brain vascular and neurodegeneration MRI markers in community-dwelling older adults. We focused on multiple MRI indices of brain white matter because S100β is predominantly found in glial cells. We also considered measures of GM and global atrophy as comparators. Notably, our results suggest that individual differences in serum S100β concentrations may be potentially informative for specific aspects of brain white matter aging. We found that higher S100β was, in cross-sectional analysis at age 73 years, significantly associated with generally poorer white matter microstructure (as indexed by *g*FA), with a small effect size (Cohen, 1992). Further investigation of tract-specific effects indicated that this association is predominantly driven by lower FA in the anterior thalamic, arcuate, cingulum, and callosal fibers.

The significant *g*FA-S100β association at age 73 years reported here contradicts some (van der Leeuw et al., 2017), but corroborates other (Milleit et al., 2016, Streitbürger et al., 2012) previous cross-sectional associations in smaller (N ≤ 102) samples. Our well-powered longitudinal design provides important new data on the coevolution of this serum biomarker with brain MRI, including several measures that had not previously been examined, such as white matter MD and markers of SVD. Given the prevalent expression of S100β in the corpus callosum (Streitbürger et al., 2012), it is notable that associations between tract-specific change and S100β change for both FA and MD in the genu of the corpus callosum were not significant. This merits further investigation in longitudinal samples over a longer period with more sampling occasions (which could also take account of nonlinear age-related trajectories).

Although there has been relatively little research on the association between S100β and age-related brain and cognitive decline, our findings that higher concentrations are related to poorer white matter FA could partly be related to deleterious effects due to systemic inflammation. Systemic inflammatory challenge reportedly elicits increased BBB permeability in humans and rodent models (Elwood et al., 2017), and there are relationships between higher inflammatory markers and lower brain metrics, including white matter markers of SVD (Aribisala et al., 2014, Corlier et al., 2018, reviewed in; Persson et al., 2014 and in; Wardlaw et al., 2013). Consequently, it will be of interest to quantify the degree to which the relationship between inflammation and cognitive decline is mediated by S100β and brain structural outcomes, as well as to identify the potential genetic and lifestyle determinants of inflammation (e.g., Corlier et al., 2018) in well-powered longitudinal designs. Taken together, our results provide some limited support for the hypothesis that both (serum markers and brain MRI) provide meaningful and overlapping biomarkers of age-related white matter degradation.

This study also provides novel information about the concentrations and stability of individual differences in serum S100β (i.e., the correlation between samples taken 3 years apart), in generally healthy older adults. These may suggest that serum S100β concentrations in the same individual may represent a relatively stable trait, although establishing this more robustly would require many more sampling occasions. We also provide information on sex differences in the context of important confounds of age, melanoma, and dementia. Significant associations between greater S100β at older ages have been reported in some studies (Nygaard et al., 1997, Schroeter et al., 2011, van Engelen et al., 1992), but were nominally negative in others (Wiesmann et al., 1998) or null (Portela et al., 2002). With respect to sex differences, our finding that females exhibited higher S100β corroborates the findings from some studies (Gazzolo et al., 2003), whereas others report the converse pattern (Nygaard et al., 1997) or no significant difference (Portela et al., 2002, Streitbürger et al., 2012, Wiesmann et al., 1998, van der Leeuw et al., 2017). Unlike the present study, those cited previously were all cross-sectional and represent a mix of single studies across very wide age ranges (neonate to 70 years old), across serum and CSF sampling (with varying sensitivity; Wiesmann et al., 1998), and comprising participants with various characteristics (healthy controls, mood disorders, those undergoing diagnostic lumbar puncture, or surgery with spinal anesthesia). Moreover, as far as the authors are aware, there is no large-scale data on the stability of individual differences in serum S100β concentrations across time in nonpathological older adults. The results reported here therefore address a substantial gap in our understanding of stability and longitudinal S100β trajectories in older community-dwelling adults.

Although it has been hypothesized that observed increases of S100β with age could reflect (1) age-related increases in myelin loss, it could also be that (2) CNS cell “turnover” remains stable, but that cellular S100β concentrations are simply higher (Nygaard et al., 1997), or that (3) S100β does not change, but that serum concentrations are driven by greater age-related BBB leakage. Our findings lend some support to the first or third interpretations. Nevertheless, it should be noted that white matter FA can be affected by multiple microstructural properties, including myelination, but also extending to axonal bore, cell membranes, microtubules, and other structures (Beaulieu, 2002, Jones et al., 2013). As such, inferences about the weak associations of S100β with any specific microstructural property of the brain's white matter should be undertaken with caution.

There are several study limitations. We note that our measure of change is based on a relatively brief (3 years) period. Although older individuals are at higher risk of brain structural changes than their younger counterparts, the brief sampling window limits the opportunity for large brain structural changes to take effect, especially because this group was broadly healthy, but fairly typical of similarly aged community-dwelling adults in Europe. Further study with a longer sampling period or a larger sample is merited to increase our ability to reliably assess these potentially subtle coupled changes, and to account for the likelihood that observed changes over time are nonlinear. On a related note, our models of latent change derived from single-indicator latent measures did not allow for the independent estimation of measurement residuals, meaning that our measures of change here should be considered as essentially difference scores (not accounting for the covariates). These analyses at only 2 time points also preclude tests of nonlinear change and of lead-lag relationships of change in brain and serum markers. We also reiterate that S100β concentrations may be influenced by a number of factors, such as exercise, melanoma, dementia, sleep apnea, depression, time of year/season, bone fractures, muscle injury, and burns (Anderson et al., 2001, Chaves et al., 2010, Harpio and Einarsson, 2004, Koh and Lee, 2014, Mohammed et al., 2001, Morera-Fumero et al., 2013, Pelinka et al., 2003, Peskind et al., 2001, Polyakova et al., 2015, Traxdorf et al., 2016), only some of which (dementia and melanoma) were accounted for in the present analyses. Our measure of PVS and its change is likely to be relatively insensitive; the rater could not be blinded to time, and the binary and disproportionate nature of visually rated PVS change mean that the estimates reported here should be interpreted accordingly. Computational methods for PVS quantification that are currently in development (Ballerini et al., 2016) may improve sensitivity to detect important aging-related changes. Finally, the narrow age range, ethnic homogeneity (all participants were White British), and relative good health of study participants limits the degree to which our findings can be generalized to groups of different ages, ethnicities, and patients. Nevertheless, the fact that these characteristics obviate such strong potential confounds in the current analysis can be viewed as an important strength.

Combined with the large sample size, longitudinal data, rich multimodal imaging parameters, same-scanner MRI acquisition, advanced and appropriate statistical modeling, and inclusion of important covariates, the present study is well-situated to test hypotheses about cross-sectional and short-term longitudinal associations between serum S100β and brain structural aging. High and increasing concentrations of serum S100β at this age is identified here as a potentially meaningful marker of poorer brain white matter health and, with further testing, risk of future dementia. These findings require replication in other well-powered healthy and pathological aging samples, and across a longer time period.

## Disclosure statement

The authors have no actual or potential conflicts of interest.
