# Supplementary material for: Longitudinal serum S100β and brain aging in the Lothian Birth Cohort 1936
Source: Neurobiol Aging. 2018 Sep;69:274–82. doi: 10.1016/j.neurobiolaging.2018.05.029 (PMC6075468; doi:10.1016/j.neurobiolaging.2018.05.029)
Supplement: Supplementary Materials [file mmc1.docx]

**Supplementary Materials**

*Table A1.* S100β intra- and inter-assay precision.

| ***Intra-assay*** | **Level 1 (0.11μg/L)** | **Level 2 (2.6μg/L)** | **Level 3 (18.4μg/L)** |
| --- | --- | --- | --- |
| **N** | 5 | 5 | 5 |
| **CoV%** | 6.4 | 3.9 | 3.6 |
|  |  |  |  |
| ***Inter-assay*** |  |  |  |
| **N** | 5 | 5 | 5 |
| **CoV%** | 10.7 | 2.2 | 3.2 |

*Note.* CoV: coefficient of variation.

*Table A2*. S100β levels in LBC1936 excluded groups.

|  | **S100β levels** | | **Sample Size** | |
| --- | --- | --- | --- | --- |
| **Group** | **Wave 2** | **Wave 3** | **Wave 2** | **Wave 3** |
| Melanoma | 0.093 (0.042) | 0.098 (0.036) | 28 | 25 |
| Dementia | 0.087 (0.042) | 0.093 (0.054) | 24 | 21 |
| Remaining group | 0.085 (0.035) | 0.092 (0.040) | 776 | 619 |

*Note.* Mean (SD) reported. Data are for participants irrespective of having provided an MRI scan.

*Table A3*. Correlations among study variables.

|  |  | 1 | 2 | 3 | 4 | 5^†^ | 6^†^ | 7 | 8 | 9 | 10 | 11 | 12 | 13 | 14 |
| --- | --- | --- | --- | --- | --- | --- | --- | --- | --- | --- | --- | --- | --- | --- | --- |
| 1 | S100β W2 | ***** | 0.585 | -0.138 | -0.125 | -0.013 | -0.008 | -0.047 | -0.112 | -0.113 | 0.130 | 0.171 | 0.152 | 0.148 | 0.110 |
| 2 | S100β W3 | <0.001 | ***** | -0.164 | -0.175 | 0.030 | 0.018 | -0.103 | -0.145 | -0.154 | 0.126 | 0.031 | 0.031 | 0.036 | -0.016 |
| 3 | TBV W2 | 0.001 | <0.001 | ***** | 0.980 | 0.123 | 0.159 | 0.103 | 0.922 | 0.893 | -0.589 | -0.085 | -0.083 | 0.000 | -0.028 |
| 4 | TBV W3 | 0.011 | <0.001 | <0.001 | ***** | 0.151 | 0.158 | 0.120 | 0.897 | 0.912 | -0.593 | -0.090 | -0.088 | -0.049 | -0.051 |
| 5 | WMH W2^†^ | 0.753 | 0.509 | 0.002 | 0.002 | ***** | 0.976 | 0.008 | -0.020 | -0.011 | -0.015 | 0.069 | 0.066 | 0.085 | 0.067 |
| 6 | WMH W3^†^ | 0.877 | 0.720 | 0.001 | 0.001 | <0.001 | ***** | 0.022 | 0.044 | 0.003 | -0.021 | 0.063 | 0.067 | 0.051 | 0.054 |
| 7 | PVS | 0.337 | 0.036 | 0.034 | 0.013 | 0.873 | 0.651 | ***** | 0.083 | 0.104 | 0.001 | -0.170 | -0.175 | -0.150 | -0.147 |
| 8 | GM W2 | 0.006 | 0.001 | <0.001 | <0.001 | 0.625 | 0.363 | 0.086 | ***** | 0.948 | -0.589 | -0.077 | -0.075 | 0.005 | -0.020 |
| 9 | GM W3 | 0.021 | 0.002 | <0.001 | <0.001 | 0.828 | 0.944 | 0.032 | <0.001 | ***** | -0.556 | -0.117 | -0.114 | -0.065 | -0.068 |
| 10 | Sex | <0.001 | 0.002 | <0.001 | <0.001 | 0.706 | 0.666 | 0.991 | <0.001 | <0.001 | ***** | 0.024 | 0.042 | -0.018 | 0.045 |
| 11 | Age S100β W2 | <0.001 | 0.448 | 0.035 | 0.062 | 0.090 | 0.190 | <0.001 | 0.056 | 0.016 | 0.492 | ***** | 0.989 | 0.924 | 0.931 |
| 12 | Age S100β W3 | <0.001 | 0.473 | 0.040 | 0.066 | 0.101 | 0.167 | <0.001 | 0.062 | 0.018 | 0.268 | <0.001 | ***** | 0.916 | 0.927 |
| 13 | Age MRI W2 | <0.001 | 0.378 | 0.994 | 0.314 | 0.055 | 0.293 | 0.002 | 0.905 | 0.179 | 0.653 | <0.001 | <0.001 | ***** | 0.993 |
| 14 | Age MRI W3 | 0.021 | 0.739 | 0.555 | 0.287 | 0.163 | 0.259 | 0.002 | 0.678 | 0.162 | 0.339 | <0.001 | <0.001 | <0.001 | ***** |

*Note.* ^†^log transformed. Pearson’s *r* reported in the upper diagonal, and *p* values in the lower diagonal. W2: LBC1936 wave 2, W3: LBC1936 wave 3, TBV: total brain volume, WMH: white matter hyperintensity volume, GM: grey matter volume; PVS: perivascular spaces.

*Table A4*. Unstandardised means and variances for the slope of study variables.

|  | **Slope Mean (SE)** | ***p*** | **Slope Variance (SE)** | ***p*** |
| --- | --- | --- | --- | --- |
| S100β | 0.005 (0.003) | 0.089 | 0.001 (0.000) | <0.001 |
| WMH^†^ | 0.248 (0.028) | <0.001 | 0.080 (0.005) | <0.001 |
| *g*FA | -0.007 (0.002) | <0.001 | 0.000 (0.000) | <0.001 |
| *g*MD | 2.717 (0.275) | <0.001 | 0.063 (0.018) | <0.001 |
| GM | -0.929 (0.101) | <0.001 | 0.916 (0.063) | <0.001 |
| TBV | -1.277 (0.130) | <0.001 | 1.531 (0.105) | <0.001 |

*Note.* Estimates (*p*-values) obtained from simple measurement models corrected for sampling lag (intra-wave sex differences) and sex – volumetric indices are corrected for ICV. FA: fractional anisotropy, MD: mean diffusivity, TBV: total brain volume, WMH: white matter hyperintensity volume, GM: grey matter volume. ^†^ log transformed.

*Table A5*. Fit statistics for S100β-global MRI structural equation models.

| **Model** | ***χ* ^2^** | **df** | ***p*** | **RMSEA** | **CFI** | **TLI** | **SRMR** |
| --- | --- | --- | --- | --- | --- | --- | --- |
| WMH | 42.629 | 28 | 0.038 | 0.023 | 0.993 | 0.988 | 0.026 |
| PVS | 39.439 | 14 | <0.001 | 0.042 | 0.963 | 0.936 | 0.032 |
| *g*FA | 284.004 | 205 | <0.001 | 0.019 | 0.977 | 0.969 | 0.035 |
| *g*MD | 322.817 | 205 | <0.001 | 0.024 | 0.970 | 0.959 | 0.048 |
| GM | 42.337 | 24 | 0.012 | 0.027 | 0.987 | 0.977 | 0.022 |
| TBV | 131.237 | 28 | <0.001 | 0.060 | 0.931 | 0.887 | 0.039 |

*Note.* FA: fractional anisotropy, MD: mean diffusivity, TBV: total brain volume, WMH: white matter hyperintensity volume, PVS: perivascular space rating, GM: grey matter volume, RMSEA: root mean square error of approximation, CFI: comparative fit index, TLI: Tucker-Lewis index, SRMR: standardized root mean square residual.

*Table A6.* Associations between baseline and 3-year change in S100β and volumetric MRI variables uncorrected for ICV.

|  | Cross-sectional  (age 73 yrs) | 95% CI  Upper Lower | Longitudinal  (age 73-76 yrs) | 95% CI  Upper Lower |
| --- | --- | --- | --- | --- |
| WMH^†^ | -0.026 (0.532) | -0.110 0.082 | 0.079 (0.105) | -0.001 0.183 |
| GM | -0.031 (0.443) | -0.120 0.052 | -0.058 (0.236) | -0.154 0.039 |
| TBV | -0.059 (0.148) | -0.121 0.039 | -0.069 (0.153) | -0.181 0.011 |

*Note.* Data are shown as standardised coefficients (*p* values), with bootsrapped 95% confidence intervals from 1000 draws. ^†^log transformed. WMH: white matter hyperintensity volume, GM: grey matter volume, TBV: total brain volume.

*Table A7*. Tract loadings for general factors of white matter fractional anisotropy and mean diffusivity.

| **Tract** | ***g*FA** | ***g*MD** |
| --- | --- | --- |
| Splenium | 0.400 | 0.395 |
| Genu | 0.531 | 0.533 |
| Arcuate | 0.681 | 0.691 |
| ATR | 0.683 | 0.669 |
| Cingulum | 0.525 | 0.611 |
| Uncinate | 0.689 | 0.694 |
| ILF | 0.510 | 0.508 |

*Note.* FA: fractional anisotropy, MD: mean diffusivity, ATR: anterior thalamic radiation, ILF: inferior longitudinal fasciculus.

*Table A8*. Fit statistics for structural equation models for tract-specific fractional anisotropy.

| **Model** | ***χ* ^2^** | **df** | ***p*** | **RMSEA** | **CFI** | **TLI** | **SRMR** |
| --- | --- | --- | --- | --- | --- | --- | --- |
| Anterior Thalamic | 55.394 | 28 | 0.002 | 0.031 | 0.973 | 0.956 | 0.025 |
| Arcuate | 55.510 | 28 | 0.001 | 0.031 | 0.977 | 0.963 | 0.027 |
| Cingulum | 53.702 | 28 | 0.002 | 0.030 | 0.975 | 0.959 | 0.022 |
| Inferior Longitudinal | 38.625 | 28 | 0.087 | 0.019 | 0.990 | 0.983 | 0.019 |
| Uncinate | 46.173 | 28 | 0.017 | 0.025 | 0.981 | 0.969 | 0.022 |
| CC Genu | 39.219 | 28 | 0.077 | 0.020 | 0.988 | 0.980 | 0.020 |
| CC Splenium | 44.051 | 28 | 0.027 | 0.024 | 0.985 | 0.975 | 0.021 |

*Note.* FA: fractional anisotropy, MD: mean diffusivity, TBV: total brain volume, WMH: white matter hyperintensity volume, PVS: perivascular space rating, GM: grey matter volume, RMSEA: root mean square error of approximation, CFI: comparative fit index, TLI: Tucker-Lewis index, SRMR: standardized root mean square residual.

*Table A9.* Fit statistics for structural equation models for tract-specific mean diffusivity.

| **Model** | ***χ* ^2^** | **df** | ***p*** | **RMSEA** | **CFI** | **TLI** | **SRMR** |
| --- | --- | --- | --- | --- | --- | --- | --- |
| Anterior Thalamic | 36.601 | 28 | 0.128 | 0.017 | 0.991 | 0.986 | 0.019 |
| Arcuate | 43.629 | 28 | 0.030 | 0.023 | 0.987 | 0.979 | 0.024 |
| Cingulum | 46.729 | 28 | 0.015 | 0.026 | 0.982 | 0.970 | 0.021 |
| Inferior Longitudinal | 36.393 | 28 | 0.133 | 0.017 | 0.992 | 0.986 | 0.018 |
| Uncinate | 45.103 | 28 | 0.022 | 0.024 | 0.984 | 0.974 | 0.022 |
| CC Genu | 40.412 | 28 | 0.061 | 0.021 | 0.987 | 0.979 | 0.020 |
| CC Splenium | 49.699 | 28 | 0.007 | 0.027 | 0.978 | 0.963 | 0.022 |

*Note.* FA: fractional anisotropy, MD: mean diffusivity, TBV: total brain volume, WMH: white matter hyperintensity volume, PVS: perivascular space rating, GM: grey matter volume, RMSEA: root mean square error of approximation, CFI: comparative fit index, TLI: Tucker-Lewis index, SRMR: standardized root mean square residual.

*Table A10.* Associations between baseline and 3-year change in S100Β and tract-specific fractional anisotropy.

|  | Cross-sectional (age 73 yrs) | 95% CI  Lower Upper | Longitudinal  (age 73-76 yrs) | 95% CI  Lower Upper |
| --- | --- | --- | --- | --- |
| ATR | **-0.155 (<0.001)** | -0.223 -0.086 | -0.060 (0.238) | -0.169 0.049 |
| Arcuate | -0.087 (0.032) | -0.170 -0.006 | -0.050 (0.337) | -0.165 0.062 |
| Cingulum | **-0.111 (0.005)** | -0.202 -0.020 | 0.033 (0.509) | -0.077 0.117 |
| ILF | -0.009 (0.815) | -0.079 0.087 | -0.073 (0.132) | -0.140 0.011 |
| Uncinate | -0.078 (0.058) | -0.146 -0.012 | -0.068 (0.193) | -0.160 0.002 |
| CC Genu | -0.047 (0.244) | -0.120 0.037 | -0.072 (0.143) | -0.158 0.006 |
| CC Splen | -0.087 (0.030) | -0.158 -0.011 | 0.027 (0.584) | -0.082 0.124 |

*Note.* Data are shown as standardised coefficients (*p* values), with bootsrapped 95% confidence intervals from 1000 draws. Bold text indicates FDR *q* < 0.05. ^a^log transformed. ^b^coefficients are for associations between visually-rated PVS change (rather than a latent change score) with S100β level and change. WMH = white matter hyperintensity volume, PVS = perivascular space rating, *g*FA = general factor of white matter tract fractional anisotropy, *g*MD = general factor of white matter tract mean diffusivity, GM = grey matter volume, TBV = total brain volume. Note that the *p*-values are calculated based on the Wald z-statistic in lavaan (which uses the SE), whereas the bootstrapped CIs are not necessarily symmettrical. Thus, in some cases, the CIs contain zero but the *p*-value is estimated as *p* > 0.05.

*Table A11.* Associations between baseline and 3-year change in S100Β and tract-specific mean diffusivity.

|  | Cross-sectional (age 73 yrs) | 95% CI  Lower Upper | Longitudinal  (age 73-76 yrs) | 95% CI  Lower Upper |
| --- | --- | --- | --- | --- |
| ATR | 0.050 (0.232) | -0.040 0.142 | 0.014 (0.792) | -0.078 0.105 |
| Arcuate | 0.021 (0.609) | -0.062 0.104 | -0.023 (0.660) | -0.120 0.092 |
| Cingulum | -0.026 (0.516) | -0.102 0.042 | -0.069 (0.158) | -0.148 0.025 |
| ILF | -0.059 (0.138) | -0.121 -0.002 | 0.017 (0.727) | -0.084 0.114 |
| Uncinate | -0.011 (0.792) | -0.101 0.070 | 0.037 (0.489) | -0.075 0.173 |
| CC Genu | -0.028 (0.493) | -0.123 0.067 | 0.053 (0.289) | -0.064 0.151 |
| CC Splen | 0.028 (0.486) | -0.054 0.097 | -0.053 (0.287) | -0.116 -0.029 |

*Note.* Data are shown as standardised coefficients (*p* values), with bootsrapped 95% confidence intervals from 1000 draws. Bold text indicates FDR *q* < 0.05. ^a^log transformed. ^b^coefficients are for associations between visually-rated PVS change (rather than a latent change score) with S100β level and change. WMH = white matter hyperintensity volume, PVS = perivascular space rating, *g*FA = general factor of white matter tract fractional anisotropy, *g*MD = general factor of white matter tract mean diffusivity, GM = grey matter volume, TBV = total brain volume. Note that the *p*-values are calculated based on the Wald z-statistic in lavaan (which uses the SE), whereas the bootstrapped CIs are not necessarily symmettrical. Thus, in some cases, the CIs contain zero but the *p*-value is estimated as *p* > 0.05.
